# Novel antidiabetic therapies in patients with peripheral artery disease: current perspective

**DOI:** 10.3389/fcdhc.2024.1517265

**Published:** 2024-11-26

**Authors:** Miodrag Janić, Viviana Maggio, Andrej Janež, Manfredi Rizzo

**Affiliations:** ^1^ Department of Endocrinology, Diabetes and Metabolic Diseases, University Medical Centre Ljubljana, Ljubljana, Slovenia; ^2^ Faculty of Medicine, University of Ljubljana, Ljubljana, Slovenia; ^3^ School of Medicine, Promise Department of Health Promotion Sciences Maternal and Infantile Care, Internal Medicine and Medical Specialties, University of Palermo, Palermo, Italy; ^4^ Ras Al Khaimah Medical and Health Sciences University, Ras Al Khaimah, United Arab Emirates

**Keywords:** peripheral artery disease, type 2 diabetes mellitus, SGLT-2 inhibitors, GLP-1 receptor agonist, GIP/GLP-1 receptor agonists

## 1 Introduction

Peripheral artery disease (PAD) is the third leading cause of atherosclerosis-related morbidity, after coronary and cerebrovascular diseases. Approximations of its prevalence are 10–26% in the general adult population and increase with age. PAD bears the burden of functional decline and major adverse limb events (MALE), consisting of chronic limb-threatening ischemia, acute limb ischemia, and major amputations. Chronic limb-threatening ischemia is associated with 20% mortality and amputations in one year. The lifetime risk of PAD varies based on traditional risk factors, including diabetes, smoking, dyslipidaemia, hypertension, and a sedentary lifestyle. Chronic inflammation, metals, air pollution, and depression also seem to play a role. Furthermore, albuminuria is related to leg amputations and retinopathy is associated with chronic limb-threatening ischemia and PAD, regardless of the duration of diabetes and hemoglobin A1c (HbA1c) levels (1). PAD also has an increased propensity not only for MALE, but also for significant major adverse cardiovascular events (MACE). Coronary artery disease is prevalent in 30–50% of patients with PAD, while the presence of polyvascular disease further increases susceptibility to MACE (2).

Diabetes significantly increases the risk of PAD, affecting 20–28% of people with diabetes. PAD is also a crucial risk factor for diabetic foot ulcers, and 50% of those with diabetic foot ulcers have PAD. The diagnosis of PAD and chronic limb-threatening ischaemia in diabetes is challenging due to atypical symptoms, particularly the absence of intermittent claudication and rest pain attributed to peripheral neuropathy, and medial artery calcification that affects the precision of non-invasive diagnostic tests. In people with diabetes, the progression of PAD differs from that of individuals without diabetes, manifesting itself in more distal arteries, affecting multiple bilateral arterial segments, and reducing collateral growth, thus increasing the risk of amputation. PAD in people with diabetes leads to worse outcomes, including non-healing foot ulcers, gangrene, amputation, and increased mortality related to cardiovascular disease. Approximately 70% of non-traumatic lower extremity amputations in the United States can be attributed to diabetes, this is disproportional to the overall prevalence of 12% (2, 3). Post-amputation mortality for people with diabetic foot ulcers is severe, with 50% dying in 5 years, comparable to many cancers (4).

As PAD increases the risk of MALE and MACE, its comprehensive and multidisciplinary management is of the highest importance, comprising of non-pharmacologic intervention (lifestyle modification: smoking cessation, supervised exercise therapy, Mediterranean diet, weight loss), pharmacologic intervention (antihypertensive therapy, lipid lowering therapy, antithrombotic therapy, glucose lowering therapy) and invasive therapy (endovascular and surgical revascularization) (2, 5). Revascularization procedures are performed to improve local limb conditions; however, people who have undergone these procedures still face a higher risk of MALE compared to their counterparts who have not undergone such procedures (2).

## 2 New antidiabetic therapies and peripheral artery disease

New antidiabetic therapies consist mainly of sodium-glucose cotransporter-2 inhibitors (SGLT2i) and glucagon-like peptide 1-receptor agonists (GLP1-RA). Representatives of these drug classes have been associated with a significant reduction in MACE in people with type 2 diabetes and increased cardiovascular risk (6). However, in the main outcome trials, most of the patients enrolled had type 2 diabetes and concomitant cardiovascular disease, but patients with PAD were underrepresented (2). Even though, based on their proven reduction in cardiovascular risk, which goes beyond mere glycaemic control, drugs of both classes with proven cardiovascular benefit are recommended by the 2023 ESC Guidelines for the treatment of cardiovascular disease in patients with diabetes and the 2024 ESC Guidelines for the treatment of peripheral arterial and aortic diseases as therapy of choice in people with type 2 diabetes and PAD to reduce cardiovascular events independent of baseline or target HbA1c and concomitant glucose lowering therapy (5, 7).

SGLT2i effectively reduce the risk of chronic kidney disease progression and benefit heart failure patients, common comorbidities seen in people with PAD, yet a meta-analysis of 20 trials did not find a significant impact on PAD incidence in people with type 2 diabetes. In contrast, US databases linked SGLT2i to a 1.65-fold increase in the risk of lower limb amputations compared to GLP-1RA. A similar signal was observed with a 1.97-fold increase in the risk of lower limb amputations for canagliflozin. It should be emphasized that this increased risk signal of amputation was only observed with canagliflozin and only in the CANVAS trial, but was not confirmed by the CREDENCE trial. A *post hoc* analysis of the CANVAS trial revealed that the majority of amputations associated with canagliflozin were minor and the risk was comparable for ischemia, infection, or dose of canagliflozin. Instead, the risk was primarily associated with previous amputations and established risk factors related to amputation, despite the absence of a specific identified etiological mechanism. However, the anticipated number of amputations was less than the number of MACE averted (8). Additionally, the meta-analysis conducted by Lin et al. also showed that patients treated with canagliflozin exhibited a modest increase in the risk of amputation and PAD compared to those who did not use SGLT2i. This increased risk was associated with greater weight loss in the treated cohort, in addition to a reduction in baseline diastolic pressure and pronounced reductions in both systolic and diastolic blood pressures, which are all potential markers of volume depletion (9). Thus, the potential underlying mechanism that contributes to the elevated risk of amputations can be attributed to diuretic-induced hypovolemia, provoked by SGLT2i, resulting in reduced perfusion of the particularly distal lower extremities. This hypoperfusion can initiate tissue necrosis, which subsequently leads to amputation. The diuretic effect in question is likely most significant during the initial phase of therapy (10). It should be mentioned that this is probably a specifical drug (canagliflozin) related effect and not a class effect, as there was also no similar signal from other SGLT2i cardiovascular outcome trials, but there was also no obvious benefit in PAD (11). However, the International Working Group on the Diabetic Foot advises against starting any SGLT2i in drug-naïve patients with diabetic foot ulcers or gangrene and suggests stopping use until healing occurs, thus carefully balancing the individual risk-benefit ratio (3).

In contrast, the GLP-1RA were associated with a 20% reduction in the risk of MALE compared to SGLT2i within the initial two years following commencement, with this outcome primarily attributed to a decreased incidence of chronic limb-threatening ischemia (12). This finding aligns with the anticipated potential of GLP-1RA due to its various putative anti-atherosclerotic mechanisms, including enhancement of systemic microcirculation, reduction of inflammation and oxidative stress, as well as improvements in endothelial function and vasodilation (13). Some are expected to be shed additionally (14). In a recent *post hoc* analysis of the LEADER and SUSTAIN-6 trials, both liraglutide and semaglutide demonstrated consistent reductions in MACE and cardiovascular efficacy regardless of the presence of PAD. Consequently, it appears that these drugs show an evident superiority in patients with PAD and type 2 diabetes compared to other glucose lowering medications with respect to PAD-related events (15). Furthermore, a benefit could emerge even in people with obesity without diabetes, since the SELECT trial showed a 20% reduction in MACE for people treated with semaglutide 2,4 mg. However, this trial included only 8.6% of patients with PAD (16).

In terms of current and emerging therapies, there is great anticipation for the SOUL trial. This event-driven, double-blind, placebo-controlled cardiovascular outcome trial evaluated the first oral GLP1-RA, oral semaglutide (14 mg once daily) compared to placebo, on the risk of cardiovascular events in people with type 2 diabetes and established atherosclerotic cardiovascular disease and/or chronic kidney disease. Among the enrolled individuals, 15.7% had symptomatic PAD, the category is not mutually exclusive to other territories of atherosclerotic disease (17). Recent announcement revealed a 14% reduction in MACE in this trial (18). Furthermore, tirzepatide, the first dual GIP/GLP-1 receptor agonist, is being evaluated in the SURPASS-CVOT trial, designed as a randomized, double-blind, active-controlled cardiovascular outcomes trial. People with type 2 diabetes with established atherosclerotic cardiovascular disease (25.3% had PAD) are subjected to a weekly subcutaneous injection of tirzepatide of up to 15 mg or 1.5 mg dulaglutide. The noninferiority for time to first MACE of tirzepatide compared to dulaglutide will confirm efficacy and safety of tirzepatied and its superiority to putative placebo and also determine whether tirzepatide produces a greater cardiovascular benefit than active comparator dulaglutide (demonstrating further superiority) (19).

And while analyzing mostly indirect data, the STARDUST trial revealed that daily injection of 1.8 mg of subcutaneous liraglutide for 6 months in people with type 2 diabetes and PAD directly increased peripheral perfusion detected by transcutaneous oxygen pressure (TcPO_2_) compared to placebo, thus confirming prevention of clinical progression of PAD in these patients (20). Furthermore, the results of the STRIDE trial are eagerly anticipated and will show whether a 52-week therapy with 1 mg of semaglutide once a week would increase the maximum walking distance on a constant load treadmill in people with type 2 diabetes and symptomatic PAD with a median pain-free walking distance of 114 m (maximum 186 m). Secondary outcomes include quality of life and cardiometabolic parameters (21). A similar trial is also still ongoing with the objective of evaluating the efficacy of liraglutide (NCT04146155) for 24 weeks on pain-free walking distance.

## 3 Discussion

The field of PAD in people with and without diabetes is not sufficiently explored to demonstrate all the possible favorable benefits of new antidiabetic therapies. Although GLP-1RA have shown definitive benefits in those with type 2 diabetes and more data is yet to come, their effects in other patients with PAD have not been validated. On the contrary, despite the proven efficacy of SGLT2i in a wide range of conditions in both populations with and without type 2 diabetes, there exists a lack of direct trials that evaluate their effectiveness in patients with PAD. With certain negative indicators, it is possible that such trials may never occur, casting a pessimistic outlook on this class of drugs within this demographic of patients. However, the advent of personalized medicine underscores the importance of tailoring approaches to each patient. Therefore, it is imperative to meticulously assess and balance risks and benefits to ensure that a beneficial drug does not exclude the patient who has much to gain from its use.
